# Physical Activity, Social Support, and the Health of Dementia Caregivers: A Scoping Review

**DOI:** 10.1002/alz70858_097214

**Published:** 2025-12-24

**Authors:** Hailey A. O'Neil, Paula C. Fletcher, Pamela J. Bryden

**Affiliations:** ^1^ Wilfrid Laurier University, Waterloo, ON, Canada

## Abstract

**Background:**

There has been an international effort to improve the quality of care available to persons living with dementia and their caregivers. As such, community‐based interventions designed to help mitigate the health effects associated with caregiving are essential. Physical activity (PA) and social support are two elements with the potential to enhance caregivers’ health and may be beneficial to include when designing and implementing interventions. Thus, to inform implementation and future research, this scoping review sought to describe and identify what is already known about PA and/or social support and dementia caregivers' physical, mental and social well‐being.

**Method:**

Following Arksey and O’Malley's (2005) framework, five databases (i.e., SPORTdiscus, CINAHL, PubMed, MEDLINE, PsychINFO) were searched in October 2022 and February 2024. Studies were selected based on a specific inclusion criterion: (1) dementia caregivers (>18); (2) community‐based PA and/or social support interventions; and (3) outcomes of overall health and well‐being. Covidence was used to organize all relevant studies, and two researchers independently extracted all data. Knowledge users (i.e., dementia caregivers & community program providers) (*n* = 4) were consulted within a focus group to confirm or refute the results based on their lived experiences.

**Result:**

Thirty‐one studies met the inclusion criteria. Ten studies examined PA; 12 studies examined social support; and 9 studies examined interventions inclusive of PA and social support. Regardless of the intervention type, the most common finding was participation led to enhanced mental well‐being. No difference in health outcomes were found between interventions delivered in‐person compared to online. Participation in joint reminiscence therapy was the only intervention to result in a decrease in health. All knowledge users agreed with this finding during the focus group and indicated reminiscence therapy may not be beneficial for all dementia caregivers.

**Conclusion:**

This review adds to our understanding of what is known about PA and/or social support and the health of dementia caregivers. It is evident participation in PA and/or social support is beneficial for the physical, mental and social well‐being of dementia caregivers. The findings from this review may help to inform the development and improvement of community‐based interventions for dementia caregivers.

